# CAS Landslide Dataset: A Large-Scale and Multisensor Dataset for Deep Learning-Based Landslide Detection

**DOI:** 10.1038/s41597-023-02847-z

**Published:** 2024-01-02

**Authors:** Yulin Xu, Chaojun Ouyang, Qingsong Xu, Dongpo Wang, Bo Zhao, Yutao Luo

**Affiliations:** 1 Key laboratory of Mountain Hazards and Surface Process, Institute of Mountain Hazards and Environment, Chinese Academy of Sciences, Chengdu, 610299 China; 2 State Key Laboratory of Geohazard Prevention and Geoenvironment Protection, Chengdu University of Technology, Chengdu, 610059 China; 3 Data Science in Earth Observation, Technical University of Munich, Munich, 80333 Germany

**Keywords:** Natural hazards, Computational science

## Abstract

In this work, we present the CAS Landslide Dataset, a large-scale and multisensor dataset for deep learning-based landslide detection, developed by the Artificial Intelligence Group at the Institute of Mountain Hazards and Environment, Chinese Academy of Sciences (CAS). The dataset aims to address the challenges encountered in landslide recognition. With the increase in landslide occurrences due to climate change and earthquakes, there is a growing need for a precise and comprehensive dataset to support fast and efficient landslide recognition. In contrast to existing datasets with dataset size, coverage, sensor type and resolution limitations, the CAS Landslide Dataset comprises 20,865 images, integrating satellite and unmanned aerial vehicle data from nine regions. To ensure reliability and applicability, we establish a robust methodology to evaluate the dataset quality. We propose the use of the Landslide Dataset as a benchmark for the construction of landslide identification models and to facilitate the development of deep learning techniques. Researchers can leverage this dataset to obtain enhanced prediction, monitoring, and analysis capabilities, thereby advancing automated landslide detection.

## Background & Summary

Landslides, which are significant natural hazards, pose a formidable challenge in mountainous regions worldwide^1,2^. The escalating effects of climate change, population growth, and urbanization have amplified the frequency and severity of landslides^3–6^. To effectively mitigate the risks associated with landslides, it is crucial to obtain a precise and comprehensive landslide inventory map that accurately records the occurrences and characteristics of landslides^7,8^. With the development of deep learning techniques, the leveraging of convolutional neural networks to assist in the generation of landslide inventory maps has emerged as the current trend. However, existing landslide datasets for deep learning exhibit several limitations that hinder the advancement of landslide identification research^9,10^. First, in terms of size, most datasets are relatively small, containing only a limited number of samples, with the largest publicly available deep learning landslide dataset consisting of 3799 images and the smallest dataset comprising only 59 images. This paucity of data restricts the ability to build robust and generalizable models. Second, the quality of the data may be questionable, as many models rely on datasets that are not publicly available or subject to review. These datasets often suffer from a low spatial resolution, rendering them unable to capture fine-grained features of landslides. Furthermore, the sampling of landslides is severely inadequate, which poses challenges for models to effectively learn the diversity of landslide occurrences. This undersampling issue is manifested in several ways: a limited coverage in terms of data from various areas, restricted sampling devices, and inadequate number of samples covering diverse landslide triggers, such as rainfall, earthquakes, and volcanic eruptions. These sample size, data quality, and diversity limitations collectively impede the development and applicability of landslide identification models. Moreover, the absence of benchmark datasets hinders comparative evaluations of landslide identification models, limiting ability of researchers to assess their strengths, weaknesses, and potential improvements^11–16^. Addressing this gap, we present the CAS Landslide Dataset, a comprehensive collection of 20,865 RGB images derived from nine distinct regions. This dataset combines imagery from unmanned aerial vehicles (UAVs) and satellites (SAT), providing diverse terrain and environmental conditions for training and evaluating landslide identification models. In the dataset creation process, we employed a rigorous quality assessment method to ensure the data integrity. Through experimental validation, we unequivocally demonstrated the effectiveness of this method. Additionally, through comparative analysis with currently available deep learning landslide datasets, we demonstrated the advantages of the CAS Landslide Dataset in terms of quantity, quality, and generalizability. These findings verified the potential of the CAS Landslide Dataset as a standardized reference dataset for training and benchmarking landslide models developed by other researchers. In other words, our dataset could serve as a standardized dataset for other researchers to train and compare the performance levels of various models. By leveraging the diversity and comprehensiveness of this dataset, researchers can develop more precise and potent models for accurately identifying landslides, thereby enhancing disaster management and risk mitigation strategies. The openly accessible CAS Landslide Dataset, with its broad geographical coverage, could enable the scientific community to advance the understanding of landslide mechanisms and contribute to reducing the impact of landslides on humans. In Table 1, we provide representative samples and corresponding labels extracted from each subdataset within the dataset. Each row corresponds to one sample, showcasing an image and the associated label from the respective subdataset.

**Table 1:** Images and Labels of Samples from the Subdatasets of the CAS Landslide Dataset.

Subdataset	Image	Label	Subdataset	Image	Label
Palu			Hokkaido Iburi-Tobu		
Lombok			Tiburon Peninsula (Sentinel)		
Tiburon Peninsula (Planet)			Mengdong		
Moxitaidi (SAT)			Moxitaidi (UAV-0.6 m)		
Moxitaidi (UAV-1 m)			Moxi town (0.2 m)		
Moxi town (1 m)			Longxi River (SAT)		
Longxi River (UAV)			Jiuzhai valley (0.2 m)		
Jiuzhai valley (0.5 m)			Wenchuan		

## Methods

### Study areas

**Fig. 1:**
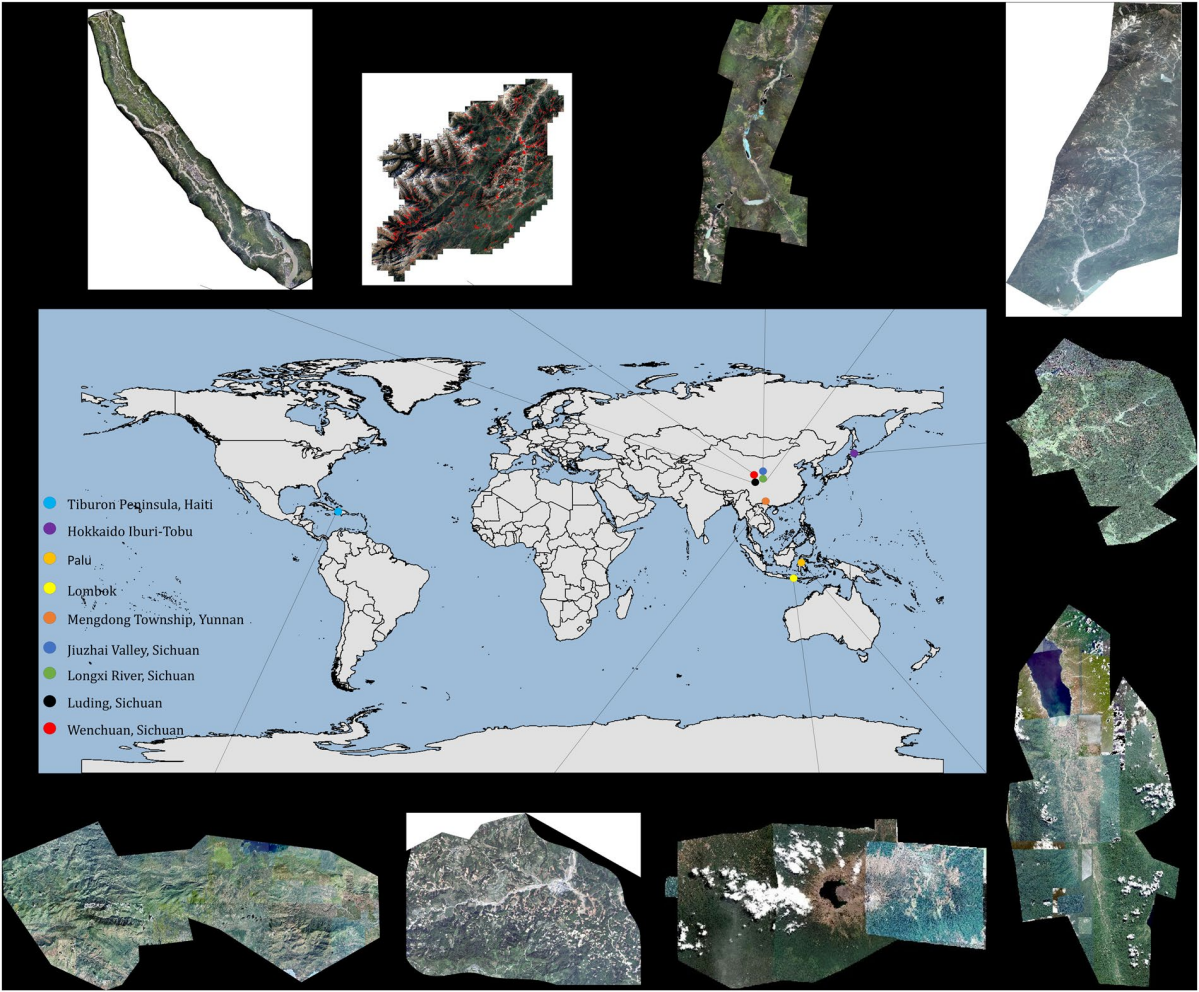
Location map of the study areas.

Our focus is on creating a standardized landslide dataset for deep learning, encompassing a diverse range of terrains, climate conditions, and vegetation cover levels, and incorporating data derived from various sources, such as UAV and satellite imagery. A location map of the study areas is shown in Fig. 1 below.

### Data acquisition

The majority of our satellite imagery is sourced from various publicly available datasets provided by different organizations and can be accessed through the Google Earth Engine (GEE) platform^17^. These include Sentinel-2A/B (SEN2)^18,19^ and Landsat^20^. Our UAV imagery is sourced from collaborative partners and can be accessed through instructions provided later. To assist users in identifying our study areas, georeferenced shapefiles (shp files) delineating each research region were incorporated in the dataset. The images of Tiburon Peninsula (Sentinel), Moxitaidi (SAT), and Wenchuan originate from Google Earth Engine. Their utilization necessitates due adherence to the stipulations outlined in the Google Earth Engine (GEE) terms and conditions, as specified by the guidelines^21^. The imagery of the Tiburon Peninsula (Planet) was sourced from Planet’s Education and Research Program^22^.The imagery of Palu, Lombok were sourced from Digital Globe Open data Program^23,24^.Hokkaido Iburi-Tobu is sourced from Geospatial information Authority of Japan^25^. The imagery of Mengdong was procured through legitimate authorization from Beijing Lanyu Fangyuan Technology Co., Ltd. For those seeking access to the raw data, it is advised to directly engage with the aforementioned company and follow the purchasing guidelines outlined on their official website^26^. The imagery of Longxi River (SAT) was procured through legitimate authorization China Centre for Resources Satellite Data and Application^27^. Furthermore, the UAV images of the Longxi River, Jiuzhai Valley, and Luding were provided by the Sichuan Geomatics Center, an essential collaborative partner of the institutions of the authors of this work. Others wishing to repeat the work or perform similar studies may approach the Sichuan Geomatics Center^28^ or access their database^29^. For information regarding the source and capture time of the subdataset, please refer to Table 2 below.

**Table 2:** Detailed Information on the CAS Landslide Dataset.

Subdataset	Amount	Acquisition Time	Source	Sensor	Ground Resolution (m)	Authorization
Palu	817	2021.01-2021.11	Digital Globe Open Data Program	WorldView2/3	5	CC-BY-NC 4.0
Lombok	436	2019.05-2019.12	Digital Globe Open Data Program	WorldView2/3	5	CC-BY-NC 4.0
Hokkaido Iburi-Tobu	1484	2018.09-2018.10	Geospatial information Authority of Japan	SAT	3	BY 4.0
Tiburon Peninsula (Sentinel)	606	2020.03-2020.06	European Space Agency	Sentinel-2/L2A	5	Non-commercial use
Tiburon Peninsula (Planet)	325	2021.09-2021.12	Planet	Planet	4	Planet education and research program
Mengdong	1155	2018.11.04	Beijing Lanyu Fangyuan Technology Co., Ltd.	SuperView-1	0.5	Image Licence
Moxitaidi (SAT)	652	2022.09-2022.10	European Space Agency	Sentinel-2/L2A	0.6	Non-commercial use
Moxitaidi (UAV-0.6 m)	984	2022.09-2022.10	Sichuan Geomatics Center	UAV	0.6	Derivative Works Licence
Moxitaidi (UAV-1 m)	483	2022.09-2022.10	Sichuan Geomatics Center	UAV	1	Derivative Works Licence
Moxi town (0.2 m)	1635	2022.09-2022.10	Sichuan Geomatics Center	UAV	0.2	Derivative Works Licence
Moxi town (1 m)	160	2022.09-2022.10	Sichuan Geomatics Center	UAV	1	Derivative Works Licence
Longxi River (SAT)	1769	2015.03-2015.12	China Centre for Resources Satellite Data and Application	GF-1	0.5	Image Licence
Longxi River (UAV)	2504	2011.03-2011.05	Sichuan Geomatics Center	UAV	0.5	Derivative Works Licence
Jiuzhai valley (0.2 m)	5925	2017.08-2017.09	Sichuan Geomatics Center	UAV	0.2	Derivative Works Licence
Jiuzhai valley (0.5 m)	1752	2017.08-2017.09	Sichuan Geomatics Center	UAV	0.5	Derivative Works Licence
Wenchuan	178	2008.11-2008.12	U.S. Geological Survey	Landsat	5	Follow the terms and guidelines by LP DAAC

### Label creation

With reference to the disclosed landslide interpretations from previous work^30–34^, in conjunction with the acquired imagery, we created labels using QGIS version 3.32.3 and LabelMe software. QGIS was utilized for its comprehensive geospatial analysis capabilities, allowing for the precise analysis of landslide-related geographic information. LabelMe, however, was employed for our dataset due to its user-friendly interface and high suitability for semantic segmentation tasks. These tools were chosen based on their capabilities and suitability for accurately interpreting landslide features within the given context.

We used the following standards to ensure the accuracy and quality of the labels:

- Reference Data:
  We referred to existing landslide inventories and published sources to cross-verify our results and ensure alignment with recognized landslide interpretations.
- Expert Input:
  Our production process involved collaboration with domain experts and geologists, whose expertise in landslide identification and analysis contributed to the generation of accurate and consistent results.
- Quality Control Measures:

We implemented stringent quality control procedures, including cross-verification of the results by multiple team members and resolution of discrepancies through discussion and consensus.

### Building the dataset

**Fig. 2:**
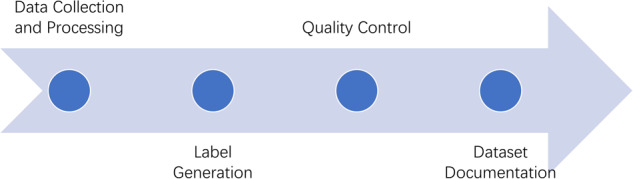
Workflow for Building the Dataset.

We cropped the images into the 512 × 512 size TIFF format, and the label files, which contain interpretations of landslides corresponding to each image, were created in the same format. Specifically, the workflow for creating the dataset is shown in Fig. 2.

When creating the dataset, we encountered various challenges, as indicated in Table 3 below: insufficient content in cropped images (image boundary)^35–37^, low proportion of target objects (label pixel proportion)^37–39^, target obstruction by cloud cover (cloud)^40,41^, and discontinuity in the image content due to image stitching (seam)^42–44^. The incorporation of problematic data exhibiting these issues into the training dataset could result in increased computational costs because more invalid data must be processed. This could also cause model accuracy reduction, as the model may overfit the invalid data and yield biased predictions on the valid data. In contrast, excluding problematic data could decrease the computational costs and improve the model accuracy to some extent^45–49^. However, the resulting model must still resolve these problematic data during actual detection, which could significantly compromise the accuracy due to the model’s lack of experience with such data, yielding a less robust model. To address these data-related challenges that arise during image cropping and labelling, we devised a rigorous screening and filtering scheme. Specifically, we first used automated metrics to identify and quantify issues such as the image boundary, target size, and occlusion percentage. Images failing to meet certain thresholds were flagged. We then manually inspected the flagged images to make the final rejection or retention decision. For example, after iterative screening of the initial SAT dataset, we filtered out approximately 1,245 problematic images, which is approximately 14% of the initial dataset. This process allowed us to create a refined dataset, as evidenced by the 1% increase in the validation accuracy over models trained on the unfiltered dataset. The experimental results in this section are detailed in the Validation of Dataset Quality Control section below.

- Image boundary
  Due to the size of remote sensing (RS) images, which often exceeds the processing capacity of neural networks in terms of resolution and storage space, preprocessing operations such as cropping and scaling are typically needed before inputting the images into the neural network for training^50,51^. In the cropping process, we encountered the issue of boundary filling. Boundary filling refers to areas in the RS images that do not cover actual objects and are typically filled with white pixels or a fixed value. To maintain the integrity of the original image information while minimizing the negative impact of excessive white pixels on the model during training, we established a threshold. Data with a proportion of filled pixels exceeding 30% were excluded, ensuring that only the most relevant and informative data were used for training purposes.
- Label pixel proportion
  Within the context of landslide detection in RS images, one frequently encounters the small-sample detection problem. The proportion of pixels representing landslide areas in the satellite images of the region is relatively low. This poses a challenge when constructing the dataset, as individual images may contain only a minute fraction of landslides. Even for human observers, identifying landslide areas becomes arduous under such circumstances. Consequently, model training can be adversely affected. To address this issue, we established a threshold to exclude data in which the proportion of labelled pixels in a single image falls below 0.1%. As such, we ensured that the dataset primarily consists of images with a more notable representation of landslide areas, enabling more effective model training.
- Cloud
  Earthquakes and rainfall events are the primary natural hazards that can trigger landslides in mountainous areas, often resulting in extensive cloud cover in postevent satellite images. Mitigating the impact of clouds on landslide identification poses a persistent challenge in this domain. To enhance the model robustness while reducing the interference of excessive poor-quality image data during training, we opted to exclude satellite image instances in which the proportion of cloud pixels exceeds 80% and the clarity of landslide pixels is compromised. This strategic decision enabled us to incorporate only high-quality image data and enhance the model effectiveness in accurately detecting landslides triggered by earthquakes and rainfall events.
- Seam
  Imaging artefacts referred to as seams denote the observed discrepancies in brightness, colour, or texture between satellite images captured at different times or locations. These artefacts stem from variations in camera angles, lighting conditions, or ground changes during image acquisition. This issue is more prevalent in historical images and satellite images depicting underdeveloped regions. To curate our dataset effectively, we carefully excluded low-quality images exhibiting severe misalignment and blurred representations of landslide regions. This careful selection process ensured that our dataset contained pertinent information while mitigating the adverse impact of image artefacts, ultimately enhancing the robustness and accuracy of our model in landslide detection.
- Manual inspection

**Table 3:** Challenges in Building the Dataset.

Image Boundary	Label Pixel Proportion	Cloud	Seam
			

After applying the automated and manual filtering procedures as detailed earlier, we conducted a meticulous visual inspection of both the retained and excluded datasets. This involved overlaying the labels onto the images and conducting a careful visual assessment to ensure the accuracy of the labels in relation to the actual features in the images. Specifically, we examined whether the labels accurately covered the corresponding landslides in the images. This thorough visual examination was crucial to validate the integrity and reliability of our dataset, thus enhancing the accuracy and quality of the data.

### Model

To assess the performance and the usability of our dataset in semantic labelling tasks, we selected several deep learning models, including three renowned models commonly used in landslide identification and a deep learning network previously proposed to reinforce landslide recognition. Specifically, these models are an FCN^52^, U-net^53^, DeeplabV3+^54^, and MFFENet^55^.

## Data Records

The CAS Landslide Dataset has been uploaded in Zenodo^56^. It is designed to be open and accessible to all landslide researchers and professionals. The data associated with this work can be accessed from the repository, which contains a project file labelled CAS Landslide Dataset, along with a README file, a study areas shp file and 16 zip files representing the different subdatasets. Each subdataset consists of three subfolders: img, label, and mask. It is important to note that in our mask files, landslide areas are labelled as 1, while non-landslide areas are labelled as 0.

Each subdataset within the dataset consists of three folders: img, label, and mask. All data within the dataset are in TIFF format and have a resolution of 512 × 512 pixels. To provide an overview of the key parameters of the dataset, they are compiled in Table 4, which is included and uploaded alongside the dataset.

**Table 4:** Geological and Environmental Characteristics of the Study Areas.

Location	Triggering Factors	Landforms	Lithology	Climate	Vegetation
Palu	Earthquake	Coastal lowlands, mountains, valleys, and rivers	Sandstone, shale, and volcanic rocks	Tropical monsoon climate with high rainfall and humidity	Rainforest, mangrove, and savanna vegetation
Hokkaido Iburi-Tobu	Earthquake	Mountains, plateaus, and coastal plains	Andesite, dacite, rhyolite, and basalt	Humid continental climate with heavy snowfall	Broad-leaved and mixed forests
Lombok	Earthquake	Mountains, hills, plains, and beaches	Andesite, basalt, and tuff	Tropical climate with distinct wet and dry seasons	Tropical rainforest, savanna, and grassland
Tiburon Peninsula, Haiti	Earthquake	Coastal plains, hills, and rugged mountains	Limestone, shale, and sandstone	Tropical climate with rainy summers and dry winters	Dry forests, mangrove, and scrubland
Mengdong township, Yunnan	Rainfall	Karst hills, canyons, and rivers	Limestone, shale, and sandstone	Subtropical monsoon climate with mild temperatures	Evergreen broad-leaved forests and bamboo
Luding, Sichuan	Earthquake	Mountainous terrain and valleys	Sandstone, shale, and limestone	Humid subtropical climate with high precipitation	Deciduous broad-leaved forests and shrubs
Longxi River, Sichuan	Rainfall	Mountains, canyons, and rivers	Sandstone, shale, and limestone	Subtropical monsoon climate with moderate rainfall	Mixed forests, bamboo, and shrubland
Jiuzhai Valley, Sichuan	Earthquake	Mountainous terrain, valleys, and lakes	Limestone, dolomite, and shale	Subarctic climate with long and cold winters	Alpine vegetation, coniferous forests, and grassland
Wenchuan, Sichuan	Earthquake	Mountainous region with rugged terrain	Sandstone, shale, and limestone	Humid subtropical climate	Deciduous broad-leaved forests, coniferous forests, and shrubs

## Technical Validation

For training purposes, the DeepLabV3+, U-net, and MFFENet models utilize ResNet50 as the underlying backbone network, and FCN utilizes VGG16 as the backbone network. In regard to the model parameter settings, our implementation utilized PyTorch as the framework, employing the SGD optimizer with a learning rate of 0.01, a momentum of 0.9, and a weight decay of 0.0005. Given that landslide identification entails a task with imbalanced data samples, we utilized the Dice loss as our loss function. Notably, the model was trained on one NVIDIA Tesla V100-SXM2 32 GB video card.

Landslide extraction from remote sensing imagery is commonly approached as a task of semantic segmentation, wherein the aim is to precisely categorize pixels into two distinct classes: foreground and background. Within this framework, the assessment of segmentation performance entails the quantification of the intersecting region, denoting the count of veritable positive (TP) pixels, and the amalgamation, signifying the cumulative sum of TP, false positive (FP), and false negative (FN) pixels. Concretely, TP corresponds to accurately identified landslide pixels, FP denotes erroneously classified landslide pixels (belong to the non-landslide), and FN represents erroneously classified non-landslide pixels (belong to the landslide). We utilize six typical metrics: namely, precision, recall, F1 score, IoU, mIoU and Overall accuracy (OA). More specifically, precision reflects the false alarm rate, recall reflects the miss alarm rate of the model. Whereas F1 takes both indices into account; therefore, a larger score indicates a better model. loU represents the overlap rate of the change class on the detection map and the ground truth. MIoU is the average IoU across all classes. It calculates the IoU for each class and then takes the mean over all classes. MIoU provides a comprehensive measure of the detection performance across categories. OA (Overall Accuracy) is the overall accuracy of pixel classification. It reflects the proportion of all samples that are correctly classified. A higher OA indicates more accurate classification results. These six metrics can be calculated as follows:

Precision:

1 $$Precision=\frac{TP}{TP+FP}$$

Recall:

2 $$Recall=\frac{TP}{TP+FN}$$

F1 score:

3 $$F1\;score=\frac{2\times {\rm{Precision}}\times {\rm{Recall}}}{{\rm{Precision}}+{\rm{Recal}}}$$

IoU:

4 $$IoU=\frac{TP}{TP+FP+FN}$$

mIoU:

5 $$mIoU=\frac{1}{n}{\sum }_{i=1}^{n}Io{U}_{i}$$

OA:

6 $$OA=\frac{TP+TN}{TP++TN+FP+FN}$$

TP: True Positives

TN: True Negatives

FP: False Positives

FN: False Negatives

n: Number of Classes

### Validation of the CAS landslide dataset

The CAS Landslide Dataset was primarily built using UAV and SAT imagery data obtained from 9 distinct regions. To validate the quality of the dataset, we followed the approach proposed by Géron A. and randomly split each subdataset into training and validation sets at a 7:3 ratio^57^. Next, we conducted model training on the carefully filtered dataset, which includes data from UAV, SAT, and combined UAV and SAT sources. The results for our datasets are listed in Table 5.

**Table 5:** Results for the Subdatasets.

UAV						
Model	Precision	Recall	IoU	F1 score	mIoU	OA
FCN	75.045%	84.016%	65.057%	86.724%	77.456%	91.468%
Unet	73.694%	86.394%	65.991%	87.136%	78.019%	91.658%
DeepLabv3+	89.289%	93.739%	84.261%	94.715%	90.142%	96.721%
MFFENet	89.326%	93.839%	84.375%	94.756%	90.214%	96.746%
**SAT**						
FCN	62.981%	84.142%	55.716%	84.391%	75.173%	94.972%
Unet	61.795%	78.550%	51.179%	82.316%	72.619%	94.410%
DeepLabv3+	74.275%	89.187%	68.137%	89.675%	82.397%	96.881%
MFFENet	74.141%	89.141%	67.998%	89.621%	82.318%	96.862%
**UAV&SAT**						
FCN	70.847%	84.014%	61.757%	85.864%	76.515%	92.848%
Unet	67.479%	82.360%	60.115%	85.311%	75.697%	92.653%
DeepLabv3+	86.128%	92.013%	80.125%	93.563%	88.316%	96.687%
MFFENet	86.133%	92.121%	80.088%	93.608%	88.299%	96.754%

In our three datasets, the FCN and Unet models attained commendable scores, with mIoU values ranging from 72% to 78% and F1 scores ranging from 82% to 87%. The intricate network models, namely, DeepLabv3+ and MFFENet, yielded impressive outcomes, exhibiting an mIoU value ranging from 82% to 90% and an F1 score ranging from 89% to 94%. These findings accentuate the robustness and potential of our datasets. Upon horizontal comparison of the three datasets, it became apparent that the UAV dataset yielded the highest scores across all models. In contrast, the satellite dataset yielded the lowest scores, suggesting that its quality may not be on par with the UAV dataset. This discrepancy in the model performance could be attributed to the lower quality of satellite imagery than that of UAV imagery. Significantly, when considering the comprehensive unification of UAV and satellite datasets, the models achieved favourable scores. This demonstrates the robustness of our dataset in the domain of unmanned aerial vehicles and satellite imagery while providing valuable data support for landslide recognition employing multisensor images. Furthermore, it facilitates the production of subsequent large datasets and the training of significant models.

### Validation of dataset quality control

In this section of the experiment, the quality control methods mentioned in the Building the dataset section are validated. The original SAT dataset used originates from an unfiltered SAT dataset, while the SAT dataset is consistent with the one used in the Validation of the CAS Landslide Dataset section, which is the dataset screened and ultimately published. The experimental results are presented in Table 6 below. The analysis of the datasets to be released and the original version revealed substantial disparities in their performance. The SAT dataset outperformed the original dataset across multiple vital metrics, including precision (74.275% vs. 72.365%), recall (89.187% vs. 88.382%), IoU (68.137% vs. 66.275%), F1 score (89.675% vs. 88.759%), mIoU (82.397% vs. 81.233%), and overall accuracy (96.881% vs. 96.457%). These outcomes indicate that the SAT dataset provides more precise and dependable labels, resulting in a superior segmentation performance. We eliminated a total of 1245 images, yet the model performance was actually improved. Specifically, the IoU metric, directly associated with landslide identification, increased by 1.862%, while the F1 score increased by 0.916%. This demonstrates the overall effectiveness of our screening method, resulting in not only computational savings but also accuracy enhancement.

**Table 6:** Results of Dataset Quality Control.

Model	Dataset	Number	Precision	Recall	IoU	F1 score	mIoU	OA
DeepLabv3+	Original SAT	8658	72.365%	88.382%	66.275%	88.759%	81.233%	96.457%
	SAT	7413	74.275%	89.187%	68.137%	89.675%	82.397%	96.881%

### Comparative experiment of the published landslide datasets for deep learning

To showcase the exceptional quality and robustness of our dataset, we compared it with previously published datasets. We carefully selected a validation set comprising 2119 images of UAV and satellite data from the region of Moxitaidi, while the remaining data were categorized into UAV and satellite classes and reconstituted as a training set. We obtained the RGB data from the Bijie Landslide Dataset^58,59^, which is a high-precision aerial imagery and interpretation dataset of landslide and debris flow disasters in Sichuan and surrounding areas (Sichuan and Surrounding Areas Landslide Dataset)^60^, HR-GLDD, which is a globally distributed high-resolution landslide dataset^61,62^, and Landslide4Sense^63^. To ensure consistency during training, we standardized the image to a resolution of 512 × 512 pixels. Table 7 presents our experimental results.

**Table 7:** Results of Dataset Comparison.

Model	Dataset	Number	Precision	Recall	IoU	F1 score	mIoU	OA
DeepLabv3+	Bijie	770	22.147%	45.389%	17.483%	59.380%	48.830%	80.977%
	Sichuan and Surrounding Areas Landslide Dataset	59	35.580%	67.124%	30.280%	69.312%	57.854%	86.295%
	HR-GLDD	1119	9.733%	35.746%	8.282%	46.577%	36.019%	64.909%
	Landslide4Sense	3799	69.222%	1.005%	1.000%	48.678%	46.080%	91.168%
	UAV	11972	71.714%	53.151%	43.806%	78.812%	68.733%	93.958%
	SAT	6762	58.984%	29.589%	24.449%	67.468%	58.061%	91.892%
	SAT + UAV	18734	66.741%	60.219%	46.319%	79.963%	69.885%	93.801%

The data presented in this table reveals unexpected findings. Notably, the dataset encompassing Sichuan and its surrounding areas, despite comprising only 59 data samples, significantly outperforms the Bijie dataset, HR-GLDD and the dataset of the AI4RS group and provides a performance that approaches that of our SAT dataset in the Moxitaidi detection task. In comparison to the three other publicly available datasets, our dataset exhibits superior performance in terms of IoU, F1 score, and mIoU. These results highlight its exceptional capability in accurately identifying landslides within the designated task area. The exceptional training outcomes of the dataset for Sichuan and its surrounding areas can be attributed to several factors. First, the training set covers a geographically similar area to the verification set, both situated in Sichuan province, China. Second, the aerial images in the training set exhibit a commendable level of quality. Among the three datasets created the SAT+UAV dataset is notable, exhibiting impressive results that show the robustness of utilizing multisource data when managing unknown images. Moreover, a comparison between the UAV and SAT datasets reveals a positive correlation between the quality of the training set and the ability to identify landslides. Interestingly, despite the inferior quality of the SAT dataset in prior baseline analysis, this experiment yields results on par with those obtained with the superior Sichuan and Surrounding Areas Landslide Dataset. This suggests that the limitations of the satellite dataset primarily stem from the quality of the images themselves. It is imperative to emphasize that, for the datasets involved in this comparison, we solely employed RGB optical images for training, without incorporating additional data such as DEM data to aid in the training process. Despite containing a total of 1785 images, it is worth noting that the HR-GLDD dataset primarily consists of 1119 images allocated to the training set, while the remaining images are divided into test and verification sets.

## Usage Notes

The CAS Landslide Dataset offers ultrahigh-resolution, multimodal, and diverse scenarios encompassing various terrains, climates, and vegetation changes. However, it is crucial to acknowledge its limitations. Specifically, the quantity of our dataset for deep learning tasks is still relatively small, and there are significant regional differences among certain subdatasets. These differences should be considered when training and utilizing the CAS Landslide Dataset to account for their potential impact on results. Furthermore, it is important to consider the limitations of the dataset, such as the spatial resolution ranging from 0.2–5 m and the data derived from SAT and UAV sources when interpreting results and evaluating the performance of the CAS Landslide Dataset.

## Data Availability

The code for cropping, generating dataset labels is publicly available: https://github.com/Aizu0/CAS-Landslide-Dataset-production-code.
